# Paradigm Shift for COVID-19 Response: Identifying High-risk Individuals and Treating Inflammation

**DOI:** 10.5811/westjem.2020.3.47520

**Published:** 2020-04-13

**Authors:** Paul Kivela

**Affiliations:** The University of Alabama at Birmingham, Department of Emergency Medicine, Birmingham, Alabama

As an emergency and wellness physician, scientist, father, and 55-year-old man, I have a keen interest in the coronavirus and the resulting COVID-19/severe acute respiratory syndrome (SARS) CoV2 illness. Based on all I have heard from the scientific community, a review of the literature, and a review of historical facts related to other epidemics, I believe we are missing some key points, particularly with regard to how we are approaching prevention of morbidity and mortality. My opinion is the virus is not killing people, but rather the immune response to the virus. Perhaps we need to shift our thinking from screening and treating signs and symptoms to add risk stratifying, measuring, and treating inflammation. I think this approach needs to be seriously vetted. It might be used to screen not just our patients, but also our physicians and nurses to determine who should be seeing patients frontline vs remotely, which patients should be admitted, and which should be sent home.

In the 1918 influenza pandemic, roughly 25% of the world’s population was infected and it caused between 50 million and 100 million deaths.^1^ If we want to have a different outcome, we have to do something different. I agree that social distancing and quarantine are both key measures to flatten the curve (Figure 1) and slow transmission so that resources are not as overwhelmed. However, we must also consider novel approaches to treat patients who have been infected or may get infected while we wait for a vaccine.

To manage these patients appropriately we must examine the cause of the morbidity and mortality associated with COVID-19. Historically, we know viral infections mostly affect young children and the elderly. This is primarily due to these populations being very susceptible to dehydration. However, this novel virus largely spares the young and preys primarily on those over 40.

This graph in Figure 2 shows the age of those discharged vs died from the first 150 cases in Wuhan, China.^2^ From the initial Chinese data, there were no deaths under age 40 with the first 150 patients. The Chinese Center for Disease Control data show the mortality rate logarithmically increased as patients reach 40 years of age^3^ (Table 1). Preliminary US and Italian data are similar with even more emphasis on older patients.^4^

Symptoms for younger people are generally mild and many are even asymptomatic. Figure 3 illustrates that the cause of death is from organ failure, usually the lungs or heart.^2^ Therefore, we must look beyond complications of dehydration as a key factor and look at the body’s immune response to this novel virus instead.

The immune response to this virus seems to be largely consistent with cytokine release syndrome (CRS/cytokine storm). CRS/cytokine storm is a severe systemic inflammatory response that can arise from multiple conditions including infectious and non-infectious diseases, including graft vs host disease, Ebola virus disease, smallpox, and even infusion reactions from certain medications. It is postulated that CRS/cytokine storm was the primary cause of mortality in the 1918 influenza pandemic, SARS, H5N1, and hantavirus outbreaks.

Signs and symptoms of CRS/cytokine storm are the same symptoms that critical COVID-19 patients are presenting with including fever, rapid respirations and heartrate, cough, shortness of breath, fatigue, loss of appetite, muscle and joint pain, nausea, vomiting, diarrhea, low blood pressure, seizures, headache, confusion, and delirium. We must go beyond treating this illness with supportive measures and also treat the inflammatory response to the virus. We have to shift our thinking as many physicians and healthcare workers are not familiar with CRS/cytokine storm.

What we know about seriously ill COVID-19 patients is that they develop a lymphopenia (low T-cell count) and bilateral pulmonary infiltrates, which is likely acute respiratory distress syndrome (ARDS/respiratory failure) and/or heart failure. Many critical patients have elevated cardiac enzymes, inflammatory markers (C-reactive protein [CRP]), and an elevated interleukin-6 (IL-6) level (Figure 4).^2^ Testing patients for the virus is probably not enough. Furthermore, we should also consider using other screening tools that measure inflammation such as CRP, IL-6, and ferritin levels^5^ so that we can identify those patients who are at higher risk of decompensating.

CRS/cytokine storm occurs when white blood cells are activated and release inflammatory signals (cytokines) that further activate more white blood cells. One of the key cytokines is IL-6, which is also involved in rheumatoid arthritis and several cancers. Traditionally, care for CRS has been supportive, addressing the symptoms of fever, oxygen, fluids, and medications to raise blood pressure. Traditionally, we have used nonsteroidal anti-inflammatory drugs and corticosteroids to treat severe CRS in people with ARDS; however, they have not been shown to have any significant effect on outcomes. Some human immunodeficiency virus medications may be promising but similarly have not yet been proven to be effective.

Biologics are likely the most useful medications in our arsenal to treat CRS/cytokine storm, as they have been shown to improve the immune response by decreasing IL-6. There are two biologic medications approved by the US Food and Drug Administration for anti-IL-6 therapy that may be useful for treating critical COVID-19 patients. Those medications include tocilizumab (Actemra), an antibody directed against the IL-6 receptor, and siltuximab (Sylvant), which is an anti-IL-6 chimeric monoclonal antibody. ACE inhibitors and ARB (angiotensin II receptor blockers) have also been shown to decrease IL-6 levels.^6^

Recent reports indicate that the US is studying azithromycin^7^ and hydroxychloroquine.^8^ Both of these medications have been found to decrease IL-6 levels. Some of these medications are being investigated and used in China,^9^ but as a healthcare worker in the US I have not heard that of many who are considering using medications.

We should also take notice that this virus is disproportionately affecting older men and post-menopausal women. It is interesting that testosterone levels decrease after age 30 in men (and after menopause for women). Patients with low testosterone have elevated IL-6 levels, higher incidences of diabetes, high rates of obesity, and higher overall mortality. The *Journal of the American Geriatric Society* found that “older age, adiposity, slower walking speed, higher disease burden and white blood cell count were associated with increased risk of IL-6 elevation over a three-year period.”^10^ A study published in the journal *Clinical Endocrinology and Metabolism* concluded that “testosterone replacement shifts the cytokine balance to a state of reduced inflammation” and decreases IL-6.^11^

There is a long list of other substances and behaviors that can also potentially decrease IL-6 including the following^12^: Vitamin D3,^13^ zinc,^14^ magnesium,^15^, probiotics,^16^ aspirin,^17^ fish oil/DHA,^18^ and resveratrol,^19^ to name a few. Conversely, diabetes, high blood sugar, high glycemic load foods, starchy foods, and potentially coffee can actually increase IL-6 levels.

So what can we do? I recommend that the public continue to adhere to recommendations set forth by government health officials, but go one step further and take steps to decrease inflammation by limiting sugary and fatty foods, exercising daily (preferably outdoors) and consider taking appropriate supplements to decrease IL-6 levels, and consider testosterone therapy especially in those individuals with low hormone levels.

I also recommend that physicians and healthcare providers continue to use supportive measures, but also consider screening by measuring inflammatory markers, risk-categorizing individuals, treating the immune response to this virus, and studying anti-inflammatory therapies. We can also possibly use inflammatory markers to help risk-stratify which providers should be working on the frontline and which should work remotely. We cannot sit by idly waiting for a vaccine and simply use supportive measures as we get overwhelmed with patients. We must be brave and consider novel approaches to identify, triage, and manage patients affected by this novel virus.

## Figures and Tables

**Figure 1:**
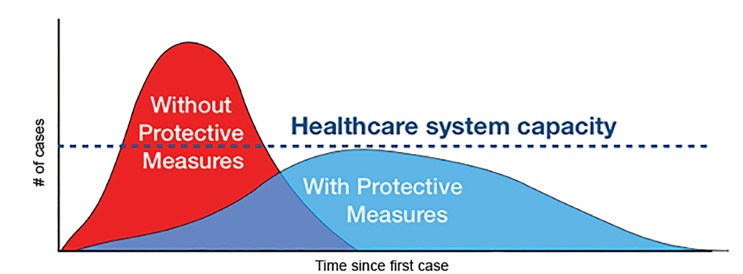
Using protective measures such as social distancing slows virus transmission and eases pressure on hospitals and providers. Adapted from the CDC/The Economist. Available at: https://www.nytimes.com/article/flatten-curve-coronavirus.html.

**Figure 2:**
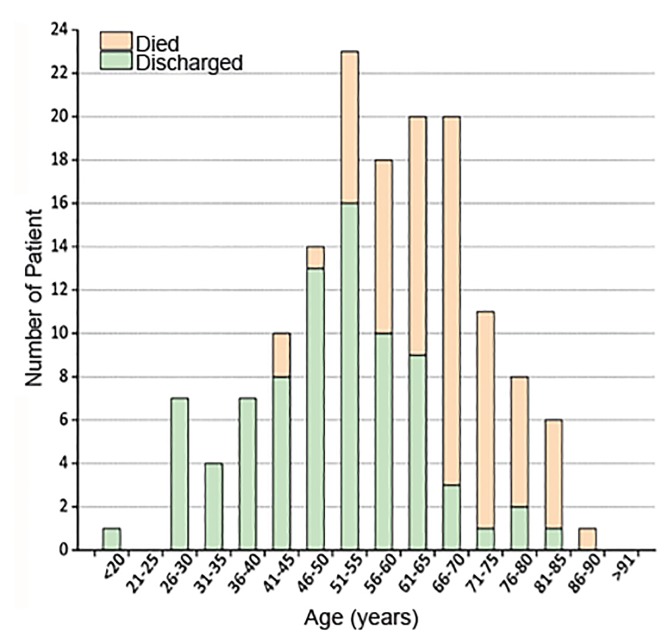
Age of discharged patients vs those who died among the first 150 cases of COVID-19 in China.^2^

**Figure 3:**
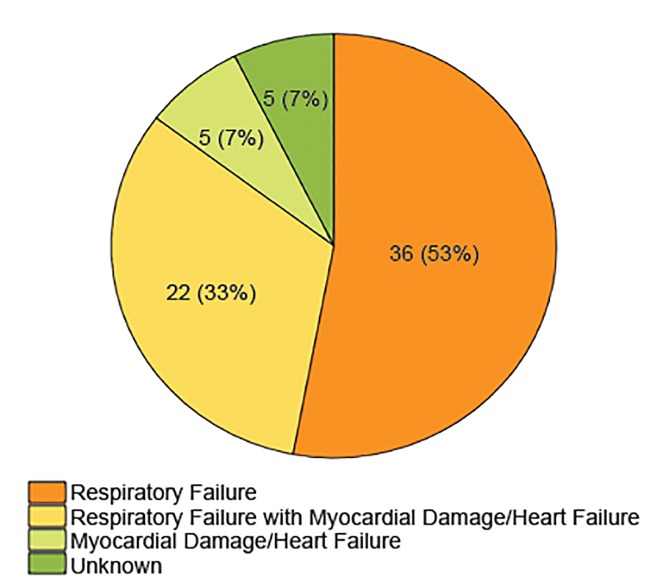
In COVID-19 patients the cause of death is primarily from organ failure, usually the lungs or heart.^2^

**Figure 4:**
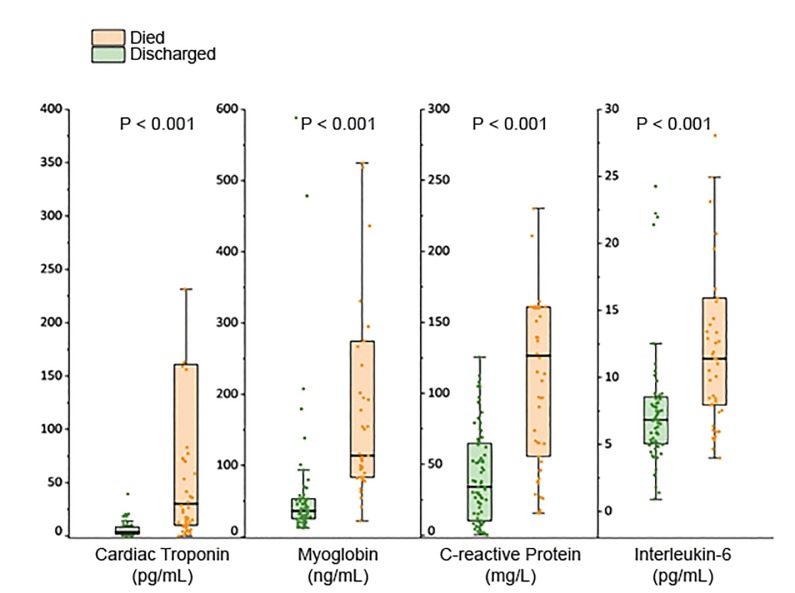
Inflammatory markers are indicative of higher mortality.^2^

**Table 1:** COVID-19 mortality rate by age.

Age Group	Mortality rate
80+ years old	14.8%
70–79 years old	8.0%
60–69 years old	3.6%
50–59 years old	1.3%
40–49 years old	0.4%
30–39 years old	0.2%
20–29 years old	0.2%
10–19 years old	0.2%
0–9 years old	None
